# Ovarian aging increases small extracellular vesicle CD81^+^ release in human follicular fluid and influences miRNA profiles

**DOI:** 10.18632/aging.103441

**Published:** 2020-06-17

**Authors:** Rosalia Battaglia, Paolo Musumeci, Marco Ragusa, Davide Barbagallo, Marina Scalia, Massimo Zimbone, Josè Maria Lo Faro, Placido Borzì, Paolo Scollo, Michele Purrello, Elena Maria Vento, Cinzia Di Pietro

**Affiliations:** 1 Department of Biomedical and Biotechnological Sciences, University of Catania, Catania 95123, Italy; 2 Department of Physics and Astronomy, University of Catania, Catania 95123, Italy; 3 Oasi Research Institue-IRCCS, Troina 94018, Italy; 4 CNR-IMM, Catania 95123, Italy; 5 IPCF-CNR, viale F. Messina 98158, Italy; 6 IVF Unit, Cannizzaro Hospital, Catania 95126, Italy

**Keywords:** reproductive aging, extracellular vesicles, microRNAs, follicular fluid

## Abstract

Ovarian aging affects female reproductive potential and is characterized by alterations in proteins, mRNAs and non-coding RNAs inside the ovarian follicle. Ovarian somatic cells and the oocyte communicate with each other secreting different molecules into the follicular fluid, by extracellular vesicles. The cargo of follicular fluid vesicles may influence female reproductive ability; accordingly, analysis of extracellular vesicle content could provide information about the quality of the female germ cell.

In order to identify the most significant deregulated microRNAs in reproductive aging, we quantified the small extracellular vesicles in human follicular fluid from older and younger women and analyzed the expression of microRNAs enclosed inside the vesicles. We found twice as many small extracellular vesicles in the follicular fluid from older women and several differentially expressed microRNAs. Correlating microRNA expression profiles with vesicle number, we selected 46 deregulated microRNAs associated with aging. Bioinformatic analyses allowed us to identify six miRNAs involved in TP53 signaling pathways. Specifically, miR-16-5p, miR214-3p and miR-449a were downregulated and miR-125b, miR-155-5p and miR-372 were upregulated, influencing vesicle release, oocyte maturation and stress response. We believe that this approach allowed us to identify a battery of microRNAs strictly related to female reproductive aging.

## INTRODUCTION

Aging can be defined as a time-dependent functional decline affecting the cells of multicellular organisms. It is characterized by a specific series of events such as genomic instability, epigenetic alterations, mitochondrial dysfunction, stem cell exhaustion and cellular senescence, affecting cellular physiology [1]. Cellular modifications related to aging are closely associated with different human pathologies, such as cancers and degenerative diseases [1]. Woman’s reproductive system shows a faster rate of aging than other body systems, which leads to an early reduction in reproductive ability [2]. In fact, when women reach 30 years old, they begin to experience a progressive loss of their ovarian reserve leading to menopause. Not only the number but also the quality of female gametes progressively decreases with maternal age. Different alterations in cytoplasmic and nuclear maturation processes have been described and the increase of non-disjunction meiotic errors probably represents one of the most representative [3, 4].

Human female germ cells develop inside a complex structure called the ovarian follicle. Several ovarian follicles undergo different maturation steps and, finally, at the preovulatory stage, tertiary or Graafian follicles consist of a single oocyte, somatic cells (granulosa and cumulus cells) and an antrum full of Follicular Fluid (FF) [5]. The ovarian follicle represents the basic reproductive unit, all of its cellular components are able to communicate with each other by gap junctions (oocyte and cumulus cells) or by FF [6]. Numerous studies have shown that advanced reproductive age determines transcriptome and proteome modifications of the cellular component of the ovarian follicles, resulting in a decrease of oocyte competence and reproductive potential [7–10]. Moreover, also FF composition, determined by different molecules such as nucleic acids, proteins and metabolites, secreted by both the oocyte and somatic follicular cells, is influenced by advanced maternal age. FF contains Extracellular Vesicles (EVs) and, specifically, the presence of exosomes has been demonstrated, first in animal models, and then in humans [8]. Exosome mediated transport is a communication mechanism between the oocyte and somatic follicular cells as well as among the different somatic cells, which is able to drive follicle development and oocyte maturation [6]. Among the different biological molecules present in FF, non-coding RNAs, as circular RNAs (circRNAs), long non-coding RNAs (lncRNAs) and microRNAs (miRNAs) have attracted the interest of various researchers [6, 11, 12]. FF miRNAs could influence follicle maturation and meiosis resumption, specific FF miRNA profiles have been associated with oocyte quality and embryo outcome [13–15]. Moreover, their altered expression has been found in different ovarian pathologies such as polycystic ovarian syndrome and premature ovarian failure [16, 17]. Many papers have demonstrated deregulated miRNA expression in oocytes, granulosa and cumulus cells in reproductive aging and two papers investigated FF miRNA expression in women of advanced reproductive age [18, 19].

Circulating miRNA profiles are commonly used to investigate different human pathologies and their use as non-invasive biomarkers or to design new therapies, based on miRNA delivery, has been focused on by many researchers in the last few years [20, 21]. In spite of this, important features of this communication system have still been poorly investigated: (i) the different number of vesicles present in a specific biological fluid; (ii) how circulating miRNA profiles could be influenced by the number of vesicles secreted in the fluid under examination.

In this paper, we investigated miRNA profiles in human FF comparing older and younger patients who underwent *in vitro* Fertilization (IVF) and verified the number of EVs according to the age of the woman. To identify the DE (Differentially Expressed) miRNAs performing a significant role in the regulation of the biological processes related to reproductive aging, we applied a normalization protocol considering the different number of vesicles in the different groups of women and compared the profiling results with the standard normalization protocol. This approach, associated with bioinformatic analysis, identified a battery of miRNAs whose deregulation is involved in the biological processes related to reproductive aging. We suggest that studies on circulating RNA profiling should consider the different vesicle distribution within biological fluids in order to identify the critical molecules involved in the regulation of cellular pathways altered in a specific pathology.

## RESULTS

### FF Small EVs increase in older women

**Figure 1:**
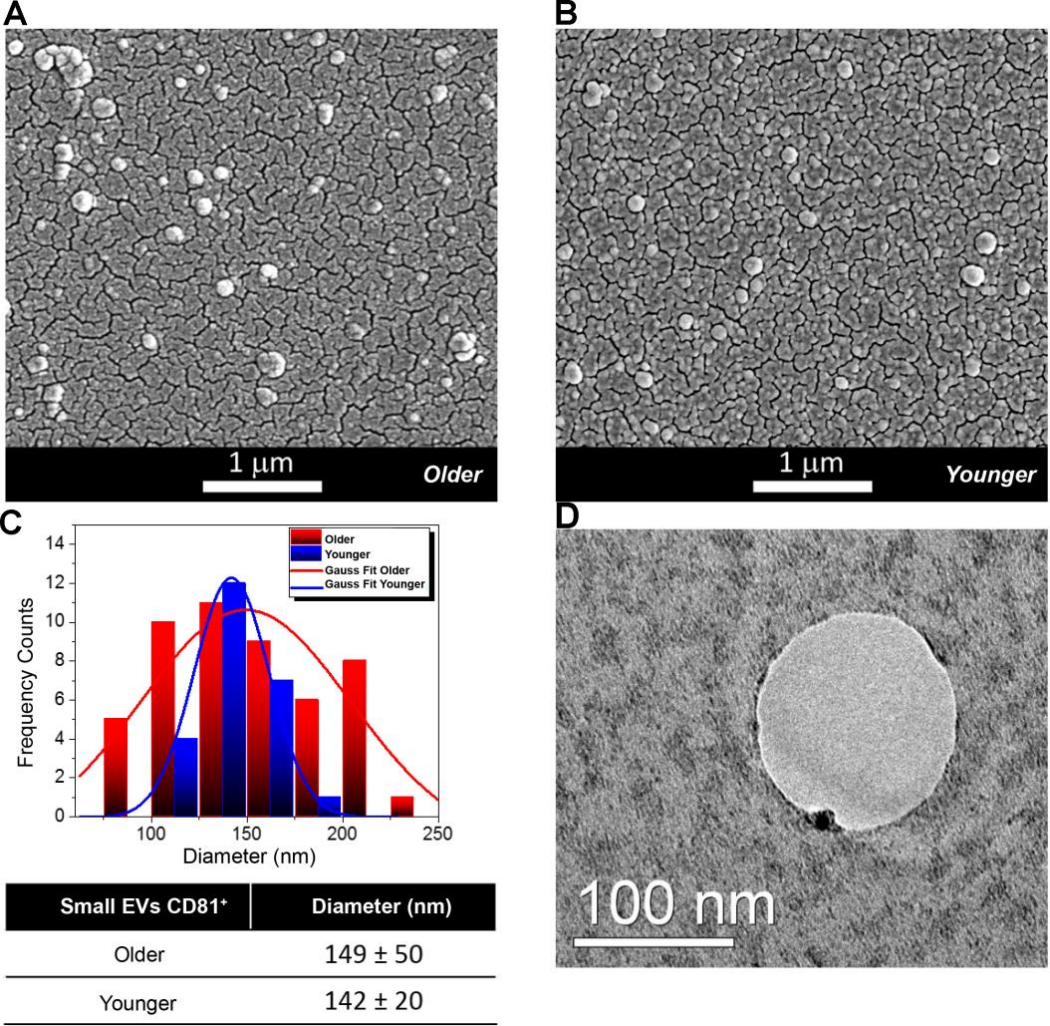
**Morphological and Molecular Characterization of Extracellular Vesicles (EVs) from Follicular Fluid (FF) of older and younger women.** (**A**, **B**) Scanning Electron Micrographs of EVs isolated from the FF of older (**A**) and younger women (**B**) showing the presence of vesicles of spherical shape with a higher abundance in FFs from older women. (**C**) Diameter distribution of EVs from FFs of older and younger women. Gauss fit of the diameters measured on SEM microscopies shows an EV average diameter of 149 ± 50 nm for FFs from older women (red curve) and of 142 ± 20 nm for those of FFs from younger women (blue trend). (**D**) The TEM analysis shows that a small Gold (Au) nanoparticle functionalized with an antibody specific for the CD81 protein marker binds to the membrane of small EVs from the FF of younger women.

The EVs from FF were characterized by Scanning Electron Microscopy (SEM), Transmission Electron Microscopy (TEM), Dynamic Light Scattering (DLS) and Nanoparticle Tracking Analysis (NTA). According to Minimal Information for Studies of Extracellular Vesicles 2018 (MISEV 2018), we will indicate our vesicles as FF small EVs, CD81^+^ [22, 23]. Figure 1A, 1B shows the SEM micrographs of EVs of spherical shape from spiked FF from older and younger women and EVs diameters (Figure 1C). The TEM analysis shows the positivity of EVs for the CD81 protein marker by using functionalized gold nanoparticles (black circles) (Figure 1D and Supplementary Figure 1).

**Figure 2:**
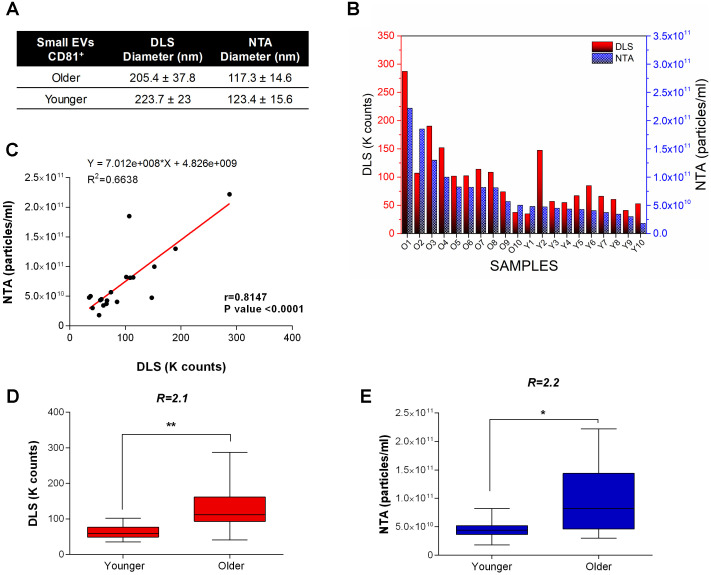
**DLS and NTA analysis.** (**A**) EV size characterization by DLS and NTA. No variation is observed for the EV size in older and younger women. A small difference in the average diameters of EVs is generally found between DLS (where the hydrodynamic radius is measured) and SEM and NTA (that measure the actual radius). (**B**) Dynamic Light Scattering (DLS) measurements and Nanoparticle tracking analysis (NTA) of small EVs from FFs of older and younger women. Scattered light Intensity, expressed as Kilocounts-per-second (Kcps), and nanoparticle concentration for FFs are shown as red and blue bars respectively. (**C**) Significant positive linear relationship between DLS and NTA measurements on FF samples. Pearson’s coefficient of correlation (r), R2, and P-value are reported. (**D**) DLS and (**E**) NTA show a significantly higher concentration of exosomes in FFs from older women. Kcps and concentration differences related to small EVs of younger and older women are reported as Box plots with Whiskers. The ratio of small EV concentrations ® between younger and older women for DLS and NTA is shown. ** P-values<0.01; * P-value <0.05.

For EV size characterization, we relied on the complimentary information acquired by several techniques based on SEM, DLS and NTA. The average diameters determined by SEM were about 149 ± 50 nm for older FF samples (red curve) and 142 ± 20 nm for younger FF ones (blue trend) (Figure 1C). Results from DLS and NTA techniques are compared in Figure 2A. No variation was observed for EV size between older and younger women. A small difference in the average diameters of EVs was found between DLS (where the hydrodynamic radius was measured) and SEM and NTA (that measured the actual radius) [24].

DLS and NTA were used to estimate the number of suspended vesicles in aqueous solutions (Figure 2B, 2C). DLS shows that the number of FF small EVs was significantly increased in the FF from older women by a factor of 2.1 with a P-value <0.01 (Figure 2D). Similarly, NTA confirmed a significantly higher concentration of small EVs in the FF from older women (1.1x 10^11^ EVs/mL) with respect to younger women (4.5 x10^10^ EVs/mL): the ratio was 2.2 with a P-value <0.05 (Figure 2E). Moreover, we found a strong dependence of the scattered light intensity (in Kilocounts-per-second, Kcps) on the nanoparticle concentration (Pearson correlation 0.8147, P-value < 0.0001) (Figure 2B).

### MiRNA expression in FF changes with female reproductive aging and is influenced by the number of FF small EVs

**Figure 3:**
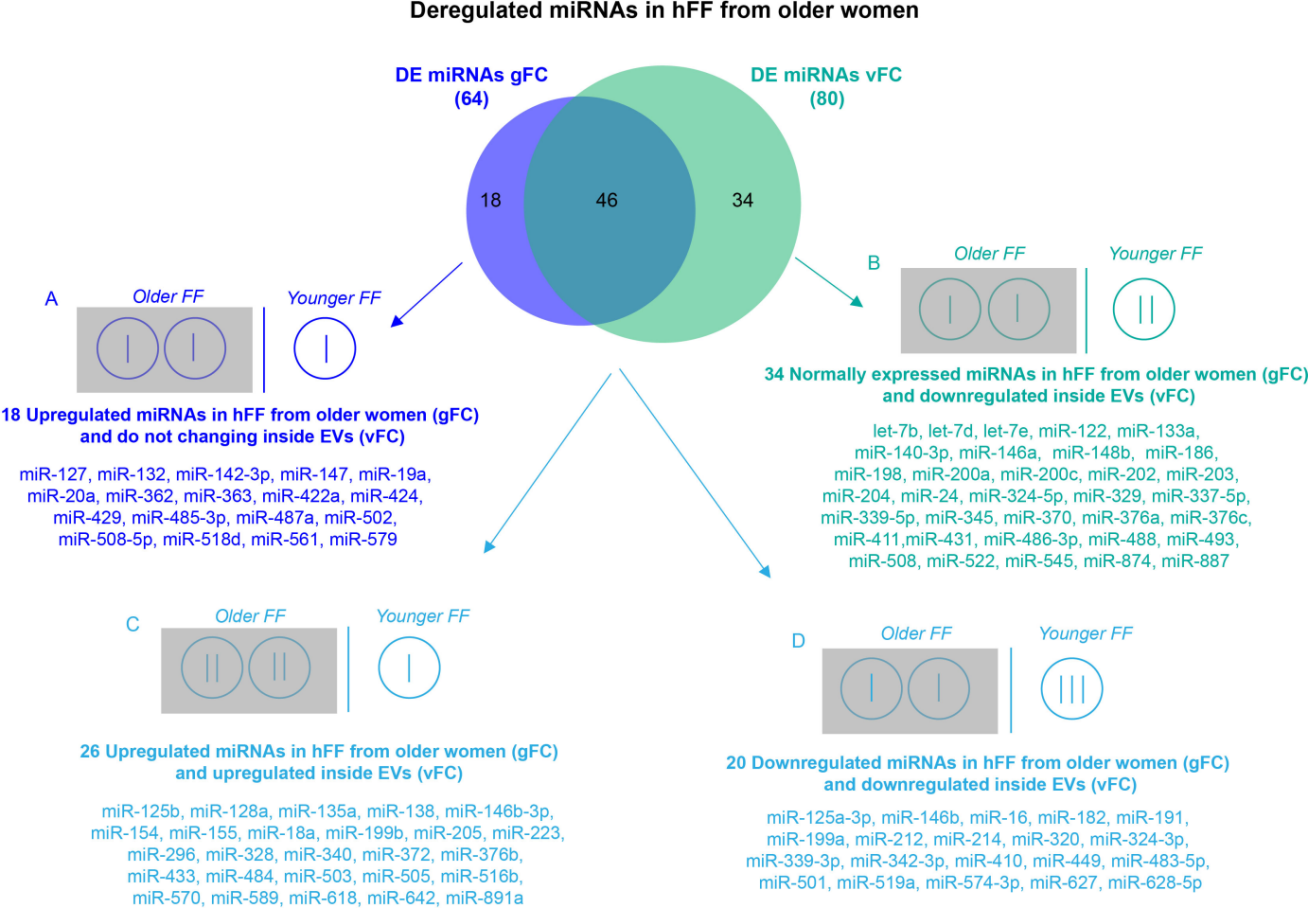
**Comparison of DE miRNA sets obtained by two normalization methods for RT-qPCR.** Differential expression analysis of miRNAs from FF EVs, comparing older women to younger women and using two normalization methods revealed 64 DE miRNAs (gFC), without considering the number of EVs, and 80 DE miRNAs (vFC) taking into account the number of vesicles. Comparison between gFC and vFC datasets identified 4 classes of DE miRNAs (**A-D**) for which we show a possible distribution (represented by vertical bars) within the individual EVs from the FF of the two groups of women according to an EV ratio of 2:1 (older vs younger).

The expression profile of the 384 miRNAs in small EVs from the FF of older and younger women, calculated by the conventional method, without considering the number of vesicles, showed 44 upregulated and 20 downregulated miRNAs in older women. After the normalization of ΔCt values for the number of EVs, the profiling results showed 26 upregulated and 54 downregulated miRNAs in FF small EVs from older women (Figure 3 and Supplementary Table 1). We reported the expression results as global Fold Change (gFC) of FF miRNAs in older women relative to younger women, and vesicle Fold Change (vFC) of FF miRNAs in exosomes of older women relative to younger women, normalized to the 2.2 particle ratio. Expression data are shown as natural logarithm (ln) of Relative Quantity (RQ). The comparison of both DE miRNA sets, related to two different normalization procedures, identified four different miRNA classes, according to the variation of their expression profiles (Figure 3). The first class consisted of 18 upregulated miRNAs in FF *in toto* and steadily expressed inside the vesicles (Figure 3A). The second class consisted of 34 downregulated miRNAs inside the vesicles, but steadily expressed in FF *in toto* (Figure 3B). The third and fourth classes included 46 miRNAs common to both normalization strategies and showing concordant variations of expression. Specifically, 26 miRNAs were upregulated and 20 miRNAs were downregulated, both in the FF and inside the vesicles from older women (Figure 3C, 3D).

### Gene ontology, pathway and network analyses

**Figure 4:**
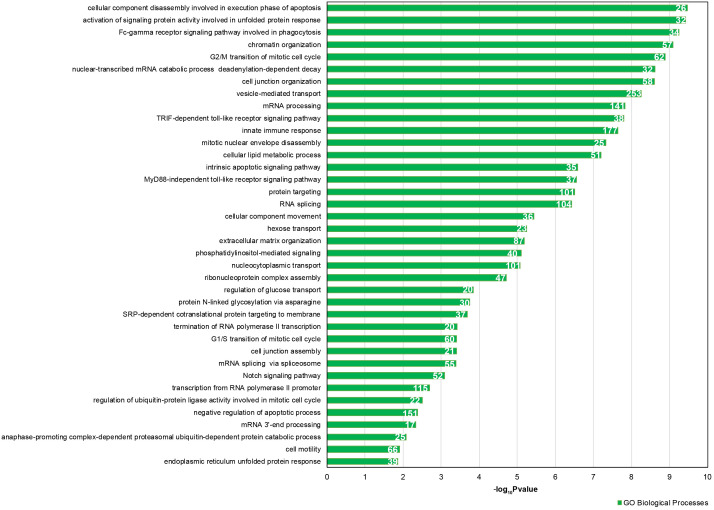
**GO enrichment analysis for 46 DE common miRNAs identified by two normalization methods.** Bar chart representing the most highly statistically enriched Gene Ontologies, in terms of Biological Processes, for DE miRNA targets in female reproductive aging. The x-axis represents the -log_10_(P-value). The number of target genes in each GO category is shown.

**Figure 5:**
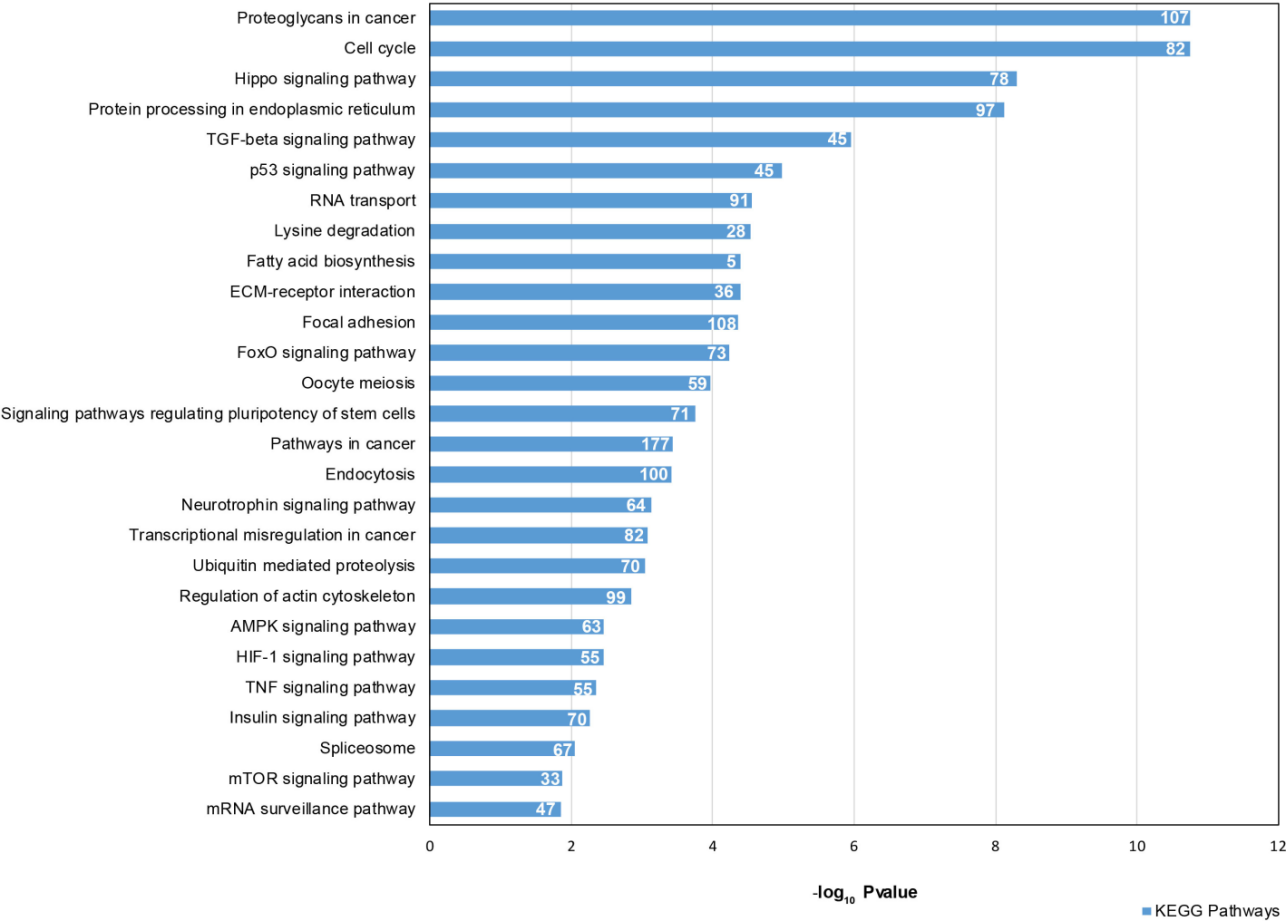
**Signaling Pathway enrichment analysis for 46 DE common miRNAs with KEGG.** The x-axis represents the -log_10_(P-value). The number of target genes in each molecular pathway is shown.

**Figure 6:**
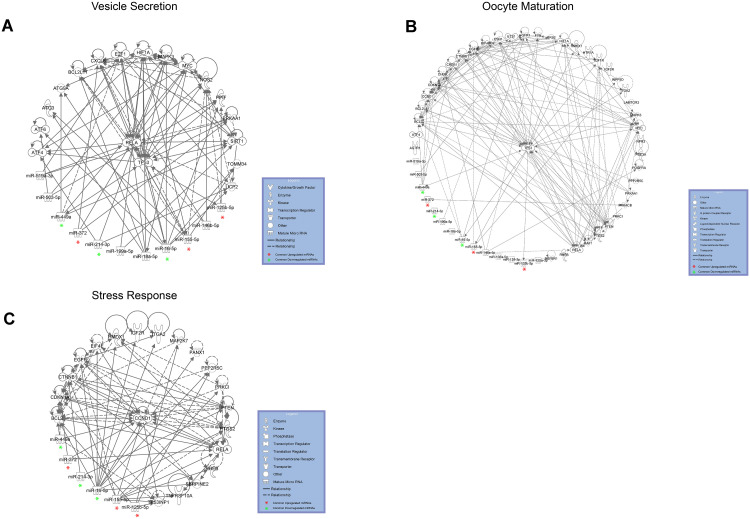
DE miRNAs with female aging control target mRNAs within complex regulatory networks of (**A**) vesicle secretion, (**B**) oocyte maturation and (**C**) stress response. DE common miRNAs are highlighted.

We focused our attention on the 46 DE common miRNAs (Figure 3C, 3D) and investigated their function for Gene Ontology (GO) classification and signaling pathway enrichment. Based on GO, most of the protein-encoding genes regulated by the identified miRNAs were involved in biological processes related to vesicle-mediated transport, mRNA processing, apoptotic signaling, nucleocytoplasmic transport, protein targeting and chromatin organization (Figure 4). Pathway analysis showed that the validated targets related to DE common miRNAs were involved in the regulation of 27 signaling pathways. Among the most significant, we found oocyte meiosis, mTOR signaling pathway, FoxO signaling pathway, p53 signaling pathway, HIF-1 signaling pathway and protein processing in the endoplasmic reticulum (Figure 5). The three networks, built on the 46 DE common miRNAs and enriched in the pathways involved in the regulation of oocyte maturation, stress response and vesicle secretion, comprise 14, 6 and 11 DE miRNAs, respectively (Figure 6). Six miRNAs are shared among the three networks: specifically, we identified miR-16-5p, miR-214-3p and miR-449a as downregulated, and miR-125b, miR-155-5p and miR-372 as upregulated (Figure 6). Moreover, TP53 is the central node in two of the three networks (Figure 6A, 6B) and RELA and CCND1 represent other central genes in these regulatory networks (Figure 6A, 6C).

## DISCUSSION

Circulating miRNAs are present in most, if not all, biological fluids: they can be free, associated with protein complexes, or enclosed within extracellular vesicles [25]. It is a common opinion that cell-free miRNAs mediate cellular communication and represent non-invasive molecular biomarkers for many human pathologies [20, 21, 25]. Many papers have investigated miRNA expression profiles in biological fluids by using different technologies: these works have demonstrated that their altered regulation is associated with specific cancer types or with other human diseases [20, 21]. The involvement of miRNAs in aging and their senescence-related changes have been demonstrated in different cellular models [26]. *In vivo,* several miRNAs have been found differentially expressed in serum or plasma during aging and in aging-related diseases [27]. In reproductive aging only two papers investigated miRNA expression in the FF from older and younger women: the authors found 1 and 4 miRNAs differentially expressed according to women’s age [18, 19]. The different distribution of miRNAs, related to specific physiologic or pathological conditions in biological fluids, could be used for diagnosis or prognosis in precision medicine, however, in many cases, the biological significance of DE miRNAs remains unknown. Moreover, an aspect still little investigated is the different vesicle quantity in fluids under specific conditions and the resulting influence on circulating miRNA profiles.

In this paper, we demonstrate an increase in the number of small EVs in the FF from older women: specifically, we found that the FF from older women contains twice as many small EVs than the FF from younger women (Figures 1, 2).

The comparison of gFC with vFC revealed four different classes of miRNAs (Figure 3). We focused our attention on the 46 miRNAs showing concordant variations of expression (Figure 3C, 3D), because they represent the miRNAs that are upregulated (Figure 3C) or downregulated (Figure 3D) both in FF *in toto* and in small EVs from older women. The higher vesicle number present in the FF of older women does not influence their altered regulation. Of course, miRNAs that are upregulated in FF *in toto* (Figure 3A) or miRNAs that are downregulated inside the small EVs (Figure 3B) could affect the follicular pathways related to aging, however, at the moment it is not clear which of the two classes could have a more important biological effect. In the first class, we found miR-424, described as upregulated in a previous study [19].

Gene Ontology, pathway and network analyses confirmed that the validated mRNA targets of the 46 identified miRNAs are involved in biological processes altered in reproductive aging, such as oocyte maturation and stress response (Figures 4–6). It is well known that oocyte quality decreases in women of advanced reproductive age. One of the most negative events is the increase of non-disjunction meiotic errors. This determines an enhanced aneuploidy rate in the embryo and the consequent reduction of fertility due to miscarriages or to the birth of newborns affected by aneuploidy [4]. Moreover, many papers have suggested the involvement of oxidative stress in oocyte aging. In the aged ovary, the balance between generation and elimination of reactive oxygen species (ROS) is impaired, which causes an increase of oxidative stress inside the ovarian follicles from aged women [28, 29].

To the best of our knowledge, this is the first report demonstrating that reproductive aging causes an increase in the number of small EVs in FF. This increase could depend on an enhanced vesicle secretion from follicular cells, as well as on a decrease of internalization mechanisms. Our data suggest that in mature ovarian follicles from aged women, vesicle release is increased. In fact, we found that some DE miRNAs are able to regulate pathways related to vesicle secretion, such as Protein processing in the endoplasmic reticulum, p53 and mTOR signaling pathways (Figure 5) [30, 31]. Moreover, the network built on vesicle secretion showed that 14 DE miRNAs out of the 46 are able to interact with mRNAs encoding key proteins in vesicle release (Figure 6).

The increase of vesicle secretion in the FF from older women could represent the result of different stresses affecting the aged ovary. Previous data demonstrated that oxidative stress increases exosome secretion in retinal pigment epithelium cells and the increased exosome release has also been reported after cisplatin treatment or after exposure to hypoxia [32–34]. Recently, it has been suggested that exosome secretion could play a role in protecting cells against intracellular stress [35, 36]. Inside the ovarian follicle from older women, granulosa cells might communicate their stress conditions to neighboring cells by increasing small EV release [36]. Thus, follicular somatic cells could enhance the antioxidant defenses protecting the follicular microenvironment and, above all, the female gamete from stress-induced damage. In a recent paper, the authors demonstrated that granulosa cells, exposed to oxidative stress, react by activating cascades of cellular antioxidant molecules, which are released into the extracellular environment through exosomes [37].

The network involved in vesicle secretion and the networks involved in oocyte maturation and stress response share six DE miRNAs: miR-16-5p, miR214-3p and miR-449a are downregulated, whereas miR-125b, miR-155-5p and miR-372 are upregulated (Figure 6). We propose that these represent important miRNAs, whose deregulation is responsible for some of the alterations detected in reproductive aging. In fact, the six miRNAs are able to control different cellular processes, all deregulated in follicles from the aged ovary. It is known that miR-155 is induced during the stress response and miR-372 has been identified as a senescence regulator [38, 39]. MiR-16-5p, miR-125b, miR-214 and miR-449a are able to control, directly or indirectly, TP53. Interestingly, the networks related to oocyte maturation and vesicle secretion have TP53 as the central node and the network related to stress response comprise molecules involved in TP53-dependent pathways, such as TP53INP1 (Figure 6). TP53 plays a major role in exosome assembly and secretion machinery: its synthesis and release under microenvironmental stress and its role in aging and senescence are well known [40–43]. A few years ago, an interesting review tagged TP53, TP63 and TP73 (the members of the TP53 family) as the guardians of maternal reproduction [44]. Maintenance of germ-line genomic integrity is a major identified function of these proteins in our species as in model organisms; different papers have demonstrated their involvement in oocyte aging and ovarian response to chemotherapy-induced stress [7, 45].

**Figure 7:**
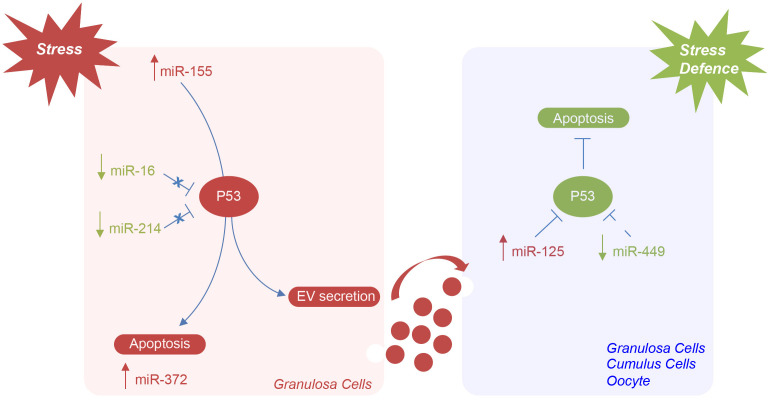
**Hypothetical model of miRNAs mediating the regulation of TP53 in the ovarian follicle in female reproductive aging.** Potential mechanisms by which specific ovarian follicle cell subpopulations can send and receive stress signaling, via EVs, in ovarian aging. MiR-155 expression is induced by stress in granulosa cells. TP53 activation, mediated by the downregulation of miR-16 and miR-214, can initiate apoptosis or lead to cellular senescence and the activation of miR-372 or cause the increase of EV secretion. The upregulation of miR-125 and the downregulation of miR-449 could represent a defense mechanism implemented by follicular cells and oocyte to repress TP53 expression and stress-induced apoptosis.

We propose that the higher number of small EVs in the FF from older women depends on an increase of vesicle secretion from somatic follicular cells, mediated by TP53, which, in turn, is activated in response to oxidative stress within the aged ovary (Figure 7). In our model, TP53 activation could be mediated by the downregulation of miR-16 and miR-214 (which act directly by binding to the TP53 mRNA) [46, 47]. TP53 activation could initiate the apoptotic process or cellular senescence in aged follicles and, at the same time, cause the increase of small EV secretion. The upregulation of miR-125 (which directly targets TP53) and the downregulation of miR-449 (which indirectly inhibits TP53 by binding SIRT1 mRNA) could represent a defense mechanism, implemented by follicular cells, to repress TP53 expression and stress-induced apoptosis [47, 48]. An antagonistic interplay in TP53 regulation has been demonstrated in cells under genotoxic stress [49].

In conclusion, we demonstrated, for the first time, the increase in the number of small EVs in the FF from older women, caused by an enhanced release from somatic follicular cells and potentially mediated by TP53 signaling pathways. By comparing the standard normalization protocol with a normalization method, which associates miRNA profiles with the number of vesicles and by using different bioinformatics tools, we identified six miRNAs deregulated in the FF from older women. Their altered profiles could influence, at the same time, oocyte maturation, stress responses and vesicle secretion. We suggest that these are among the most important miRNAs, whose deregulation is responsible for the alterations involved in reproductive aging: accordingly, they could be used as non-invasive biomarkers of oocyte quality and also as potential targets of personalized therapies. Finally, we highlight the importance of the connection between miRNA profiles and vesicle quantity in biological fluids in order to detect as DE only the most significant molecules.

## MATERIALS AND METHODS

### Patients

This comparative study was carried out on 50 healthy women (without any ovarian pathologies) known to have male-dependent primary infertility, who underwent intracytoplasmic sperm injections (ICSI) at the IVF Unit, Cannizzaro Hospital, Catania, Italy. All Patients included in the study were women 28-40 years old, who were grouped into two age categories: 25 women older than 38 years (group I) and 25 women younger than 35 years (group II) (Control group).

We excluded women aged between 35 and 38, as they represent the category with the greatest variability. Even if chronological age represents the most important factor influencing the female reproductive potential, the reproductive aging, determined by both genetic and environmental factors, varies considerably among individuals. Patients with endometriosis, polycystic ovaries, ovarian insufficiency and metabolic syndromes were excluded from the study; likewise, heavy smokers and overweight women were also excluded to avoid any limiting factors in female fertility that could have affected sample quality. Our research was carried out according to the Declaration of Helsinki; all patients included in IVF programs signed informed consent for the use of samples, prior to the study. The study was exempted from Institutional Review Board approval because patients were included in the IVF program and the FF used in our experiments represents discarded material.

### FF collection

FF samples were collected from patients who had been treated with GnRH agonists (Triptorelin or Buserelin) to induce multiple follicular development, followed by ovarian stimulation with recombinant follicle-stimulating hormone (FSH) and human menopausal gonadotropin (hMG). Stimulation was monitored using serum E2 concentrations and ultrasound measurement of follicle number and diameter. When follicles had reached a diameter >18 mm and serum E2 concentration per follicle reached 150–200 ng/L, ovulation was induced with 10,000 IU of hCG. Transvaginal ultrasound-guided aspiration of ovarian follicles was performed 34–36 h after hCG injection. FF samples were centrifuged for 20 min at 2,800 rpm at 4°C to remove residual follicular cells and any blood traces; the supernatant was immediately transferred into a clean polypropylene tube and stored at -20°C for further analysis. Samples with massive blood contamination were excluded from further analysis. The FF of individual follicles was kept separated until decumulation of the oocytes. Only FF samples, in which nuclear mature oocytes (metaphase II) had been identified, were used.

We collected FFs from 15 older and 15 younger women for miRNA profiling. Specifically, we prepared 6 pools (3 older and 3 younger), each pool consisted of 5 individual fluids. From each pool, we purified the vesicles, extracted the RNA and performed Real-Time quantitative PCR. For SEM, TEM, DLS and NTA, we used 20 FFs, 10 from older and 10 from younger women. We purified the vesicles from each sample, resuspended them in PBS and made all the analyses individually.

### Small EV purification

Forty mL of total FF, for pooled samples, and ten ml of FF diluted 1:4 in sterile Phosphate Saline Buffer (PBS), for single samples, were centrifuged at 300 x g for 15 min at 4°C to pellet debris. The supernatant was transferred to ultracentrifuge tubes and ultracentrifuged at 16,500 x g for 30 min at 4°C, followed by filtration through a 0.2-μm syringe filter. Finally, EVs were pelleted by ultracentrifugation at 120,000 x g for 70 min at 4°C in a Beckman Optima L-100 XP ultracentrifuge using a Ti70 rotor (Beckman Coulter). We refer to vesicles sedimenting at 100,000 g as “small EVs” according to Misev 2018 [22, 23]. EV pellets were resuspended in 1 mL PBS for vesicle characterization or directly lysed in Trizol (Invitrogen) for RNA isolation.

### Scanning electron microscopy

The resuspended small EV pellets from FF (10 μl) from both older and younger women were fixed in 100 μl of 3% formaldehyde-0.1 % glutaraldehyde in 0.1 M phosphate buffer overnight at 4°C. A drop of suspension (5 μl) was layered onto a sterile cover glass coated with 0.1% poly-L-Lysine, post-fixed in 1% osmium tetroxide (Merck, Darmstadt, Germany) in the same buffer for 1h at 4°C and washed in phosphate buffer. After dehydrating in graded ethanol and critical point drying, the samples were sputtered with a 5nm gold layer using an Emscope SM 300 (Emscope Laboratories, Ashford, UK) and then observed with a SUPRA 25 ZEISS microscope, at a working distance of 3-5 mm and in low accelerating voltage conditions of 2kV by using an in-lens detector. All the SEM micrographs were then analyzed by ImageJ software for image processing to measure the EV diameter statistics (https://imagej.nih.gov/ij/index.html).

### Transmission electron microscopy

The resuspended small EV pellet (10 μl) of FF was fixed in 100 μl of 3% formaldehyde-0.1 % glutaraldehyde for 3h at 4°C. A drop (5 μl) of the above suspension was deposited onto a copper-coated nickel grid and dried for 20 min (Electron Microscopy Sciences, Fort Washington, PA, USA) to absorb EVs. The grids, washed in PBS, were negatively stained with 4% uranyl acetate for 5 min. For immune electron microscopy labelling, the grids, with absorbed EVs side down, were rinsed for 2x2 min with PBS and transferred into a TBS (Tris-buffered saline pH 7.4) solution containing 1% BSA (bovine serum albumin) (TBS/BSA) for 10 min at 20 °C. Then the grids were incubated in 5% BSA blocking solution for 1.30 h at 20 °C, rinsed with PBS, and incubated in a humid chamber overnight at 4°C with a mouse monoclonal antibody CD81 (Santa Cruz Biotechnology, Heidelberg, Germany) in a dilution 1:50 with TBS/BSA. After washing for 3x3 min with TBS/BSA, the grids were stained with a 10 nm gold-labeled secondary antibody anti-mouse IgG (Sigma Aldrich) in a dilution 1:5 with TBS/BSA for 1h at 37°C in the dark. The grids were rinsed 2x2 with TBS/BSA, 2x2 with water and fixed with 1.5% glutaraldehyde in PBS for 10 min at room temperature. After rinsing again with water, the grids were post-stained with 4% uranyl acetate for 5 min and allow to air dry. Negative controls were prepared in the absence of primary antibodies, but with secondary antibody-conjugate. Observations were carried out using a JEOL JM transmission electron microscope operating at 200 kV.

### Dynamic light scattering

The analysis of the scattered light fluctuations was performed by the intensity auto-correlation function. DLS investigations were performed by an in-house made apparatus consisting in a 660 nm diode laser, a photomultiplier detector mounted on a rotating arm and a BI-9100 AT hardware correlator (Brookhaven Instruments Corporation) and operating in single-photon counting regime. Intensity autocorrelation function was measured by using the fluctuation of the scattered intensity at 90° with respect to the forward direction. The laser power ranged between 15 and 50 mW. All measurements were undertaken with a laser intensity of 50 mW at 25 °C. Analysis of the scattered light fluctuation was performed by the intensity autocorrelation function [50].

### Nanoparticle tracking analysis

NTA was used for particle counting and size measurements, based on their light scattering in correlation to their Brownian motion tracking in a liquid. Unlike DLS, NTA is independent of the particle density or their refractive index: this allows for the calculation of a size distribution profile of small particles with a diameter of approximately 10-1000 nm in a liquid suspension injected with a syringe pump. For the analysis, the samples were properly diluted (1:100) in sterile PBS to reach the optimal volume for NTA. Measurements were carried out by using a Nanosight NS300 system (Malvern Instruments Company, Nanosight, and Malvern, UK) coupled with a Blue 488 nm laser illumination in order to visualize and track the scattered light particles through a high sensitivity sCMOS camera over multiple frames by image processing, performed at room temperature ranging from 24.6–24.9°C. Sample analysis was conducted for 10 min under the following camera settings and processing conditions: Shutter 1300, Gain 512, camera level 16, NTA 3.2 Dev Build 3.2.16 and Detection Threshold 4.

### MicroRNA profiling by TaqMan low-density arrays

Total RNA from small EV CD81^+^ was extracted with Trizol (Invitrogen), according to the manufacturer’s instructions. Before precipitating the RNA with isopropyl alcohol, 20 μg RNase-free glycogen (Invitrogen) was added to the aqueous phase and the samples were stored for 16h at −80°C. RNA pellets were dissolved in RNase-free water and quantified by Qubit (Invitrogen). About 25 ng of total RNA were retrotranscribed and preamplified. Amplified cDNA products were diluted in 75 μL of RNase-free purified water and loaded onto microfluidic cards of the TaqMan Human MicroRNA Array Av2.0 (Applied Biosystems). Quantitative RT-PCR reactions were performed on a 7900HT Fast Real-Time PCR System (Applied Biosystems) as follows: 94.5 °C for 10 min, followed by 40 amplification cycles of 97°C for 30 sec and 59.7°C for 1 min.

### Expression data analysis

MiRNA expression profiles were analyzed using real-time RQ Manager software v1.2 (Applied Biosystems). Only miRNAs having Ct values below 35 were considered as expressed. To normalize miRNA profiling data, median and average expressions of the plate were calculated. Differentially Expressed (DE) miRNAs were identified by SAM (Significance of Microarrays Analysis) (https://statweb.stanford.edu/~tibs/SAM/), applying a two-class paired test among ΔCt of older and younger samples (controls) by using a P-value based on 100 permutations; imputation engine: K-nearest neighbors (10 neighbors); false discovery rate < 0.15. We considered as DE miRNAs only those resulting from the two normalization methods after at least two SAM tests. DE miRNA expression changes were calculated by applying the 2^−ΔΔCT^ method according to a previously published paper [51]. Expression data in the Results section are reported as gFC (global Fold Change) for all values. Considering that, according to NTA, the ratio of vesicles between the FF from older and younger women is 2.2 (see results), to normalize miRNA profiles using the number of vesicles and detect the significant DE miRNAs after this further normalization, we calculated 2^-ΔCt^ / 2.2 values relative to older women.

These last values together with the 2^-ΔCt^ values relating to the younger women were analyzed by SAM. DE miRNAs were selected as already described. Then, we calculated the new FC values (vesicle FC or vFC) related to DE miRNAs detected by SAM applying the following formula:

$$2^{-\Delta Ct\left(older\right)}\text{/}2^{-\Delta Ct\left(younger\right)}=2^{-\Delta \Delta Ct}.$$

Where 2^-ΔCt (older)^ represents the 2^-ΔCt (older)^ obtained with the standard normalization divided by vesicle ratio (2.2).

### MiRNA function enrichment analysis

Gene Ontology (GO) and Kyoto Encyclopedia of Genes and Genomes (KEGG) pathway analysis for common DE miRNAs were carried out with Diana-miRPath v3.0 (http://snf-515788.vm.okeanos.grnet.gr/) selecting for validated targets retrieved from Tarbase. DE miRNAs were analyzed for GO enrichment in terms of the Biological Process categories applying a P-value cut-off of 0.05. The FDR method was implemented to select the biological pathways with a threshold of significance defined by P < 0.05 and a microT threshold of 0.8. The resultant common DE miRNAs were analyzed using Ingenuity Pathway Analysis (IPA, http://www.ingenuity.com), (QIAGEN Inc.) to identify molecular interactions mediated by miRNAs in relation to aging. IPA analysis was conducted by filtering for experimentally validated interactions (from Tarbase) as well as highly predicted miRNA targets and microRNA-related Expert Findings, manually curated from published literature by Ingenuity scientific experts, and using the Tool Connect. After linking the miRNAs with their target mRNAs, we selected signaling pathways according to oocyte meiosis, stress response and vesicle secretion. In detail, apoptosis (PTEN), cell growth-proliferation and development (Gap junction, mTOR and PI3K/AKT signaling pathways), growth factor signaling (ERBB signaling pathway), and Intracellular II messenger (cAMP and ERK/MAPK signaling) were explored for oocyte meiosis. Successively, cell growth (mTOR and PI3K/AKT signaling pathways), cell stress (ATM and HIF signaling pathways) and cell toxicity (Mitochondrial dysfunction, NFK Band P53 signaling pathways) were analyzed for the stress response. Finally, protein processing in the endoplasmic reticulum, cellular stress and Sirtuins pathways were evaluated for vesicle secretion.

## Supplementary Material

Supplementary Figure 1

Supplementary Table 1
