# Supplementary material for: Ovarian aging increases small extracellular vesicle CD81+ release in human follicular fluid and influences miRNA profiles
Source: Aging (Albany NY). 2020 Jun 17;12(12):12324–41. doi: 10.18632/aging.103441 (PMC7343446; doi:10.18632/aging.103441)
Supplement: Supplementary Figure 1 [file aging-12-103441-s002..pdf]

SUPPLEMENTARY FIGURE

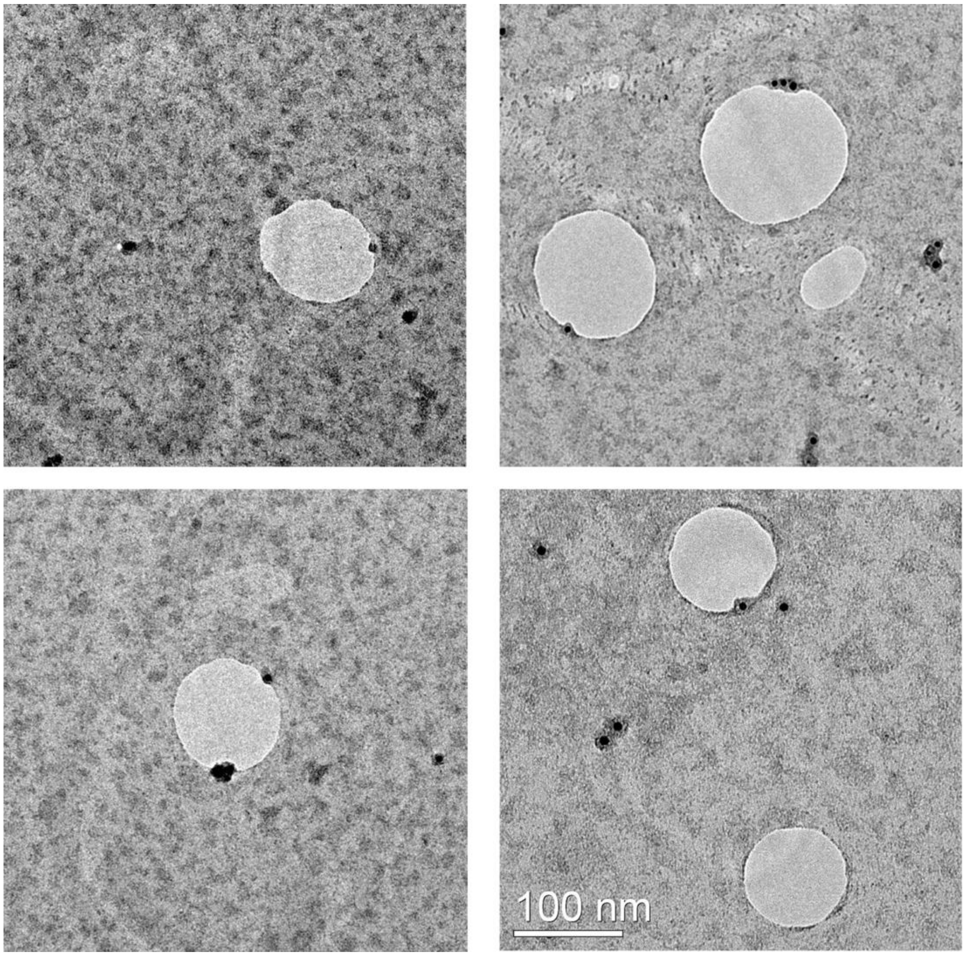

Supplementary Figure 1. TEM micrographs showing small Gold (Au) nanoparticles functionalized with an antibody specific for the CD81 protein marker binding to the membrane of small EVs from the FF of younger women.
