# Supplementary material for: Ovarian aging increases small extracellular vesicle CD81+ release in human follicular fluid and influences miRNA profiles
Source: Aging (Albany NY). 2020 Jun 17;12(12):12324–41. doi: 10.18632/aging.103441 (PMC7343446; doi:10.18632/aging.103441)
Supplement: Supplementary Table 1 [file aging-12-103441-s001..pdf]

SUPPLEMENTARY TABLE

Supplementary Table 1. Differentially expressed (DE) miRNAs according to gFC (global Fold Change) and vFC (vesicle Fold Change) normalization methods. DE miRNAs are indicated in bold.

DE miRNAs	gFC (LN RQ)	±SD	DE miRNAs	eFC (LN RQ)	±SD
let-7b	-1.09	0.21	**let-7b**	-1.87	0.21
let-7d	/	/	**let-7d**	-1.30	0.15
let-7e	/	/	**let-7e**	-1.47	0.35
let-7f	/	/	let-7f	/	/
miR-122	/	/	**miR-122**	-1.84	0.31
**miR-125a-3p**	-2.58	0.10	**miR-125a-3p**	-3.37	0.10
**miR-125b**	2.09	0.15	**miR-125b**	1.30	0.15
**miR-127**	1.58	0.08	miR-127	/	/
**miR-128a**	4.83	0.21	**miR-128a**	4.04	0.21
**miR-132**	1.82	0.05	miR-132	1.03	0.05
miR-133a	-0.97	0.18	**miR-133a**	-1.76	0.18
**miR-135a**	4.85	0.14	**miR-135a**	4.06	0.14
**miR-138**	4.89	0.31	**miR-138**	4.10	0.31
miR-140-3p	-0.93	0.04	**miR-140-3p**	-1.72	0.04
**miR-142-3p**	2.37	0.17	miR-142-3p	/	/
miR-145	/	/	miR-145	-0.47	0.09
miR-146a	-0.93	0.06	**miR-146a**	-1.72	0.06
**miR-146b**	-1.38	0.06	**miR-146b**	-2.17	0.06
**miR-146b-3p**	5.20	0.47	**miR-146b-3p**	4.42	0.47
**miR-147**	3.30	0.54	miR-147	/	/
miR-148b	/	/	**miR-148b**	-6.35	0.94
miR-150	/	/	miR-150	-0.81	0.14
**miR-154**	4.55	0.29	**miR-154**	3.76	0.29
**miR-155**	7.12	0.08	**miR-155**	6.33	0.08
miR-15b	/	/	miR-15b	-1.17	0.09
**miR-16**	-2.15	0.06	**miR-16**	-2.94	0.06
**miR-182**	-5.79	0.47	**miR-182**	-6.57	0.47
miR-186	/	/	**miR-186**	-1.55	0.42
**miR-18a**	6.11	0.57	**miR-18a**	5.32	0.57
**miR-191**	-2.19	0.07	**miR-191**	-2.98	0.07
miR-193b	/	/	miR-193b	/	/
miR-195	/	/	miR-195	-0.75	0.14
miR-196b	/	/	miR-196b	-1.01	0.16
miR-198	/	/	**miR-198**	-4.50	1.35
**miR-199a**	-3.27	0.32	**miR-199a**	-4.06	0.32
**miR-199b**	4.88	0.68	**miR-199b**	4.09	0.68
**miR-19a**	1.23	0.10	miR-19a	/	/
miR-200a	-0.79	0.12	**miR-200a**	-1.58	0.12
miR-200c	-0.99	0.13	**miR-200c**	-1.78	0.13
miR-202	-1.01	0.05	**miR-202**	-1.80	0.05
miR-203	-0.63	0.11	**miR-203**	-1.42	0.11
miR-204	-0.77	0.11	**miR-204**	-1.55	0.11
**miR-205**	4.20	0.54	**miR-205**	3.41	0.54
**miR-20a**	2.01	0.04	miR-20a	/	/
**miR-212**	-2.40	0.16	**miR-212**	-3.19	0.16
**miR-214**	-1.71	0.08	**miR-214**	-2.50	0.08
miR-218	/	/	miR-218	-0.93	0.14
miR-222	/	/	miR-222	-0.87	0.11
**miR-223**	2.05	0.05	**miR-223**	1.26	0.05
miR-24	/	/	**miR-24**	-1.31	0.13
miR-25	/	/	miR-25	-0.47	0.10
miR-28-3p	/	/	miR-28-3p	-1.03	0.14
**miR-296**	5.15	0.95	**miR-296**	4.36	0.95
miR-29a	/	/	miR-29a	-0.88	0.10
miR-302a	/	/	miR-302a	/	/
miR-31	/	/	miR-31	-1.05	0.23
**miR-320**	-1.98	0.09	**miR-320**	-2.77	0.09
miR-323-3p	/	/	miR-323-3p	-1.05	0.10
**miR-324-3p**	-5.27	0.03	**miR-324-3p**	-6.06	0.03

miR-324-5p	/	/	**miR-324-5p**	-1.67	0.15
**miR-328**	3.18	0.14	**miR-328**	2.39	0.14
miR-329	/	/	**miR-329**	-4.39	0.25
miR-337-5p	/	/	**miR-337-5p**	-1.23	0.09
**miR-339-3p**	-1.73	0.06	**miR-339-3p**	-2.52	0.06
miR-339-5p	-1.14	0.29	**miR-339-5p**	-1.92	0.29
**miR-340**	5.43	0.29	**miR-340**	4.64	0.29
**miR-342-3p**	-1.59	0.19	**miR-342-3p**	-2.38	0.19
miR-345	/	/	**miR-345**	-1.40	0.09
**miR-362**	4.01	0.36	miR-362	/	/
**miR-363**	3.80	0.24	miR-363	/	/
miR-370	/	/	**miR-370**	-1.67	0.54
**miR-372**	3.50	0.12	**miR-372**	2.71	0.12
miR-373	/	/	miR-373	/	/
miR-374	/	/	miR-374	-0.35	0.09
miR-376a	-0.95	0.08	**miR-376a**	-1.74	0.08
**miR-376b**	3.40	0.43	**miR-376b**	2.61	0.43
miR-376c	/	/	**miR-376c**	-1.46	0.20
miR-382	/	/	miR-382	-1.02	0.07
**miR-410**	-1.34	0.12	**miR-410**	-2.13	0.12
miR-411	-0.65	0.09	**miR-411**	-1.43	0.09
**miR-422a**	1.58	0.20	miR-422a	/	/
**miR-424**	2.16	0.33	miR-424	/	/
miR-425-5p	/	/	miR-425-5p	-1.14	0.09
**miR-429**	4.25	0.57	miR-429	/	/
miR-431	/	/	**miR-431**	-1.34	0.31
**miR-433**	5.45	0.23	**miR-433**	4.66	0.23
**miR-449**	-1.51	0.06	**miR-449**	-2.30	0.06
miR-454	/	/	miR-454	-1.18	0.11
**miR-483-5p**	-2.67	0.07	**miR-483-5p**	-3.46	0.07
**miR-484**	2.13	0.12	**miR-484**	1.34	0.12
**miR-485-3p**	3.57	0.25	miR-485-3p	/	/
miR-486-3p	/	/	**miR-486-3p**	-2.37	0.29
**miR-487a**	4.09	0.19	miR-487a	/	/
miR-488	/	/	**miR-488**	-2.10	0.56
miR-493	-1.05	0.21	**miR-493**	-1.83	0.21
**miR-501**	-3.17	0.23	**miR-501**	-3.96	0.23
**miR-502**	3.01	0.92	miR-502	/	/
**miR-503**	2.70	0.30	**miR-503**	1.91	0.30
**miR-505**	5.52	0.68	**miR-505**	4.73	0.68
**miR-508-5p**	6.25	0.39	miR-508-5p	/	/
miR-508	/	/	**miR-508**	-1.20	0.06
miR-509-5p	/	/	miR-509-5p	-0.58	0.15
**miR-516b**	4.38	0.31	**miR-516b**	3.59	0.31
**miR-518d**	3.07	0.49	miR-518d	/	/
**miR-519a**	-11.17	0.31	**miR-519a**	-11.96	0.31
miR-522	/	/	**miR-522**	-6.53	1.23
miR-532-3p	/	/	miR-532-3p	-1.14	0.17
miR-545	/	/	**miR-545**	-1.97	0.29
**miR-561**	3.51	0.95	miR-561	/	/
**miR-570**	3.09	0.10	**miR-570**	2.31	0.10
**miR-574-3p**	-2.31	0.10	**miR-574-3p**	-3.10	0.10
**miR-579**	3.35	0.78	miR-579	/	/
**miR-589**	4.33	0.55	**miR-589**	3.54	0.55
**miR-618**	7.44	0.84	**miR-618**	6.65	0.84
**miR-627**	-1.96	0.32	**miR-627**	-2.75	0.32
**miR-628-5p**	-1.72	0.17	**miR-628-5p**	-2.51	0.17
**miR-642**	2.75	0.50	**miR-642**	1.96	0.50
miR-652	/	/	miR-652	-1.15	0.25
miR-654	/	/	miR-654	/	/
miR-660	/	/	miR-660	-0.61	0.08
miR-744	/	/	miR-744	-0.67	0.06
miR-874	/	/	**miR-874**	-5.80	1.40
miR-887	/	/	**miR-887**	-1.85	0.64
**miR-891a**	4.68	0.32	**miR-891a**	3.89	0.32
